# Supplementary material for: MetaRibo-Seq measures translation in microbiomes
Source: Nat Commun. 2020 Jun 29;11:3268. doi: 10.1038/s41467-020-17081-z (PMC7324362; doi:10.1038/s41467-020-17081-z)
Supplement: Supplementary file 10 — Supplementary Data 7 [file 41467_2020_17081_MOESM10_ESM.zip › File2/Confidence_High_Taxonomy/111082_out.krona.html]

Javascript must be enabled to view this page.

members
magnitude
magnitudeUnassigned
count
unassigned
taxon
rank

111082\_out

5

superkingdom
5
2

1239
phylum
5

186801
5
class

186802
5
order

186803
family
5

5
genus
33042

species
5
33043

SRS014855\_contig\_number\_1659SRS015854\_contig\_number\_549SRS097958\_contig\_number\_19542SRS098571\_contig\_number\_45074SRS144714\_contig\_number\_23378
